# Optimization of Hydrolysis Conditions for the Production of Angiotensin-I Converting Enzyme-Inhibitory Peptides and Isolation of a Novel Peptide from Lizard Fish (*Saurida elongata*) Muscle Protein Hydrolysate

**DOI:** 10.3390/md10051066

**Published:** 2012-05-18

**Authors:** Shanguang Wu, Jianhua Sun, Zhangfa Tong, Xiongdiao Lan, Zhongxing Zhao, Dankui Liao

**Affiliations:** 1 School of Chemistry and Chemical Engineering, Guangxi University, Nanning, Guangxi 530004, China; Email: wusg1974@163.com (S.W.); sunjhmll@gxu.edu.cn (J.S.); bioche@gxu.edu.cn (Z.T.); lanxiongdiao@163.com (X.L.); zzxx@gxu.edu.cn (Z.Z.); 2 Department of Pharmacy, Liuzhou Medical College, Liuzhou, Guangxi 545006, China; 3 Guangxi Key Laboratory of Petrochemical Resources Processing & Process Intensification Technology, Nanning, Guangxi 530004, China

**Keywords:** ACE-inhibitory peptide, lizard fish, enzymatic hydrolysis, response surface methodology, isolation

## Abstract

Lizard fish (*Saurida elongata*) muscle protein was hydrolyzed using neutral protease to produce protein hydrolysate (LFPH), and the hydrolysis conditions were investigated using response-surface methodology. The optimum conditions for producing peptides with the highest angiotensin-I converting enzyme (ACE)-inhibitory activity were the following: enzyme-to-substrate ratio of 10,000 U/g, temperature of 48 °C, pH 7.0, and hydrolysis time of 2 h. Under these conditions, the ACE-inhibitory activity of LFPH and the degree of hydrolysis were 84% and 24%, respectively. A novel ACE-inhibitory peptide was isolated from LFPH using ultrafiltration, Sephadex G-15, and high-performance liquid chromatography. The amino acid sequence of the ACE-inhibitory peptide was identified as Ser-Pro-Arg-Cys-Arg (SPRCR), and its IC_50_ was 41 ± 1 µM.

## 1. Introduction

Hypertension is considered to be the most common chronic disease and a major risk factor for cardiovascular disease, a main cause of death worldwide [1,2]. Angiotensin-I converting enzyme (ACE) is one of the main regulators of blood pressure functioning by converting angiotensin-I into the potent vasoconstrictor, angiotensin-II, and catalyzing the degradation of the potent vasodilator, bradykinin [3]. ACE inhibitors (ACEI), a new class of antiangiotensive drugs, are effective in inhibiting the formation of angiotensin-II [4]. Synthetic ACE inhibitors, such as captopril, enalapril, alacepril, and lisinopril, have been developed as antihypertensive medicine and are widely used in the treatment of hypertension and heart failure in humans. However, some side effects caused by these synthetic drugs have been reported [5,6,7]. Natural ACE inhibitors derived from food proteins may have safety advantages over synthetic inhibitors, and a number of them have been found effective in decreasing the blood pressure of hypertensive rats and humans [8,9,10,11].

Many ACE-inhibitory peptides have been discovered from the enzymatic hydrolysates of various protein-rich foods, such as soy sauce, fish sauce, sake, soybeans, and milk. Among various sources, marine organisms have been widely used in the search for ACE-inhibitory peptides [12,13]. Most peptides that inhibit ACE are reportedly characterized by their relatively short sequences containing 2 to 12 amino acids [14]. ACE is a zinc metallopeptidase consisting of two catalytic domains called the *N*-terminal and *C*-terminal. Each domain is catalytically independent, and binding to zinc is crucial for enzymatic activity [15]. Studies of structure-activity relations among different ACE-inhibitory peptides suggest that the most potent and specific of them have similar structures, and binding to ACE is strongly influenced by the *C*-terminal sequence of the peptide. Hydrophobic residues, such as proline, lysine, or arginine, are the preferred amino acids at the *C*-terminal residue and are very important for ACE-inhibitory potency [16,17]. 

Response surface methodology (RSM) is a useful technique for exploring the relationship between several variables and generating a mathematical model to predict the values of the response variables. RSM has been successfully applied to optimize the hydrolysis conditions for producing ACE-inhibitory peptides from food proteins [18,19,20].

The lizard fish (*Saurida elongata*) is a small marine fish that lives in tropical and subtropical seas [21]. In the Guangxi province of China, lizard fish production is estimated at over 120,000 tons per year, but most of it is used as animal feed. As a rich source of protein, this fish could also be a valuable raw material for producing bioactive peptides for the treatment of diseases. 

In the present study, RSM was used to optimize the hydrolysis conditions of lizard fish, including enzyme-to-substrate ratio (E/S), hydrolysis pH, and hydrolysis temperature to obtain the most powerful ACE-inhibitory peptides. Furthermore, an ACE-inhibitory peptide was isolated from the lizard fish protein hydrolysate by ultrafiltration, Sephadex G-15, and high-performance liquid chromatography (HPLC).

## 2. Results

### 2.1. Response Surface Analysis

The optimization of enzymatic hydrolysis conditions was applied to determine the optimal values of the independent variables (temperature, E/S, and pH), which would give the maximum ACE-inhibitory activity. 

The response of DH and ACE-inhibitory activity (IP) were evaluated in CCD. The results obtained after running 20 trials according to CCD are presented in Table 1. The response of these three dependent variables to hydrolysis conditions, interactive terms, and probability values (*p*-values) are shown in Table 2 and Table 3. The effects with *p*-values lower than 0.05 indicated the statistical validity and significance of the DH and IP models. 

**Table 1:** Experimental design and results of the CCD.

No.	Temperature (°C)	E/S	pH	DH (%)	IP (%)
	X_1_	X_2_	X_3_	Y_1_	Y_2_
1	−1	−1	−1	18.73	74.66
2	1	−1	−1	19.68	76.23
3	−1	1	−1	28.48	79.52
4	1	1	−1	26.14	75.52
5	−1	−1	1	17.15	71.81
6	1	−1	1	16.76	62.10
7	−1	1	1	23.19	79.00
8	1	1	1	19.36	72.22
9	−1.68	0	0	19.69	78.00
10	1.68	0	0	16.44	70.79
11	0	−1.68	0	14.58	68.73
12	0	1.68	0	25.91	79.86
13	0	0	−1.68	23.92	79.41
14	0	0	1.68	20.34	75.33
15	0	0	0	22.39	84.04
16	0	0	0	24.30	84.96
17	0	0	0	24.29	83.20
18	0	0	0	23.81	80.08
19	0	0	0	24.08	84.30
20	0	0	0	23.98	83.16

ACE-inhibitory activity (IP), degree of hydrolysis (DH), enzyme-to-substrate ratio (E/S).

**Table 2:** Regression coefficients and their *p*-values for the linear regression model to predict the degree of hydrolysis of lizard fish muscle protein.

Source	Sum of Squares	Mean Square	*F*	*p* Prob > *F*
Model	256.02	28.45	24.75	<0.0001
X_1_-Temperature	8.98	8.98	7.81	0.0189
X_2_-E/S	141.15	141.15	122.78	<0.0001
X_3_-PH	37.37	37.37	32.51	0.0002
X_1_X_2_	5.66	5.66	4.92	0.0508
X_1_X_3_	1.00	1.00	0.87	0.3727
X_2_X_3_	7.16	7.16	6.23	0.0317
X_1_^2^	44.86	44.86	39.02	<0.0001
X_2_^2^	14.23	14.23	12.38	0.0056
X_3_^2^	1.54	1.54	1.34	0.2737
Residual	11.50	1.15		
Lack of Fit	8.91	1.78	3.44	0.1006
Pure Error	2.59	0.52		
Total	267.52			

*R*^2^ = 0.9570; adjusted *R*^2^ = 0.9184.

**Table 3:** Regression coefficients and their *p*-values for the linear regression model to predict ACE-inhibitory activity of lizard fish muscle protein hydrolysates.

Source	Sum of Squares	Mean Square	*F*	*p* Prob > *F*
Model	631.83	70.20	20.22	<0.0001
X_1_-Temperature	70.56	70.56	20.32	0.0011
X_2_-E/S	118.10	118.10	34.02	0.0002
X_3_-PH	55.98	55.98	16.12	0.0025
X_1_X_2_	0.88	0.88	0.25	0.6258
X_1_X_3_	24.71	24.71	7.12	0.0236
X_2_X_3_	21.64	21.64	6.23	0.0316
X_1_^2^	162.01	162.01	46.66	<0.0001
X_2_^2^	165.45	165.45	47.65	<0.0001
X_3_^2^	76.39	76.39	22.00	0.0009
Residual	34.72	3.47		
Lack of Fit	20.02	4.00	1.36	0.3715
Pure Error	14.70	2.94		
Total	666.55			

*R*^2^ = 0.9479; adjusted *R*^2^ = 0.9010.

Table 2 shows that X_1_, X_2_, and X_3_ were the most significant ones affecting the DH. The interactions between the different factors significantly influenced the response variable (DH), except the interaction between X_1_ and X_3_. 

As shown in Table 3, the independent variables X_1_, X_2_, and X_3_ had a significant effect on IP. The interactive effects X_1_*X_3_, X_2_*X_3_ on IP were significant. 

The coefficient of determination (adjusted *R*^2^) was used to check the fit of the models. The adjusted *R*^2^ values corresponding to DH and IP are 0.9184 and 0.9010, respectively. The adjusted *R*^2^ values were high, demonstrating that the two models were well-adapted to the responses and indicating the variability in the responses could be explained by the models (Equations 1 and 2). The lack of fit was not significant in both model equations, which, further validates the models.

Equations 1 and 2 describe the correlation between the variables and the response (DH and IP), respectively.

Y_1_ = 23.77 − 0.81X_1_ + 3.21X_2_ − 1.65X_3_ − 0.84X_1_X_2_ − 0.35X_1_X_3_ − 0.95X_2_X_3_ − 1.76X_1_^2^ − 0.99X_2_^2^ − 0.33X_3_^2 ^ (1)

Y_2_ = 83.32 − 2.27X_1_ + 2.94X_2_ − 2.02X_3_ − 0.33X_1_X_2_ − 1.76X_1_X_3_ + 1.64X_2_X_3_ − 3.35X_1_^2^ − 3.39X_2_^2^ − 2.30X_3_^2 ^ (2)

where Y_1_ and Y_2_ are the dependent variables (response variable) to be modeled; X_1_ is the variable temperature; X_2_ is the variable E/S; and X_3_ is the variable pH. 

### 2.2. Effect of Temperature, E/S, and pH on the Response Value

**Figure 1:**
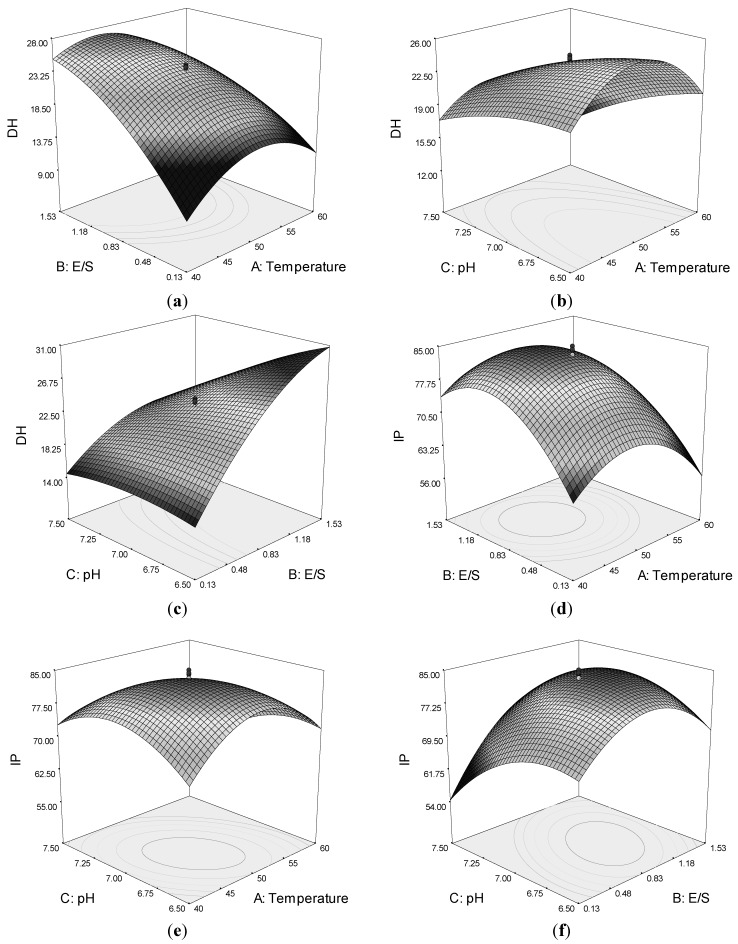
Response surface plots showing the interaction between variables on the degree of hydrolysis (DH) of lizard fish muscle protein and ACE-inhibitory activity (IP): (**a**) effect of temperature and enzyme-to-substrate ratio (E/S) on DH; (**b**) effect of pH and temperature on DH; (**c**) effect of pH and E/S on DH; (**d**) effect of temperature and E/S on IP; (**e**) effects of pH and temperature on IP; and (**f**) effects of pH and E/S on IP.

Quadratic response surfaces and regression coefficients were used to study the effects of various parameters and their interactive effects on DH and IP. The response surfaces for DH and IP were drawn as three-dimensional plots of two factors, whereas the other factors were kept constant. Figure 1a,c shows that by increasing the E/S, the DH increased. However, a maximum DH is observed around temperature 45–50 °C and pH of circa 7.0. The DH was also affected by the interaction of pH and temperature (Figure 1b).

The response surface plot for ACE-inhibitory activity as function for interaction of temperature and E/S as variables indicated a progressive increase in IP up to 10,000 U/g E/S and temperature of circa 50 °C (Figure 1d). However, a decrease in ACE-inhibitory activity was observed with a further increase of both variables. 

The effect of pH and temperature on ACE-inhibitory activity is displayed in Figure 1e. The results indicated that the ACE-inhibitory activity of hydrolysate increased with increasing temperature and pH up to an optimum point, beyond which a decrease in IP was observed for the process variables. The effect of pH and E/S is illustrated in Figure 1f, which shows that they had an interactive effect. The maximum IP value was also observed at a temperature of around 45–50 °C and pH of 7.0. The results suggested that an increase in some variables would promote the DH, but would not result in higher ACE-inhibitory activity. 

To obtain the maximum ACE-inhibitory activity of the hydrolysates, the model was optimized using Design Expert^®^ 7.0 by setting the maximum IP value (Y_2_) as the goal. The optimum conditions were temperature at 48 °C, E/S at 10,000 U/g, and pH at 7.0. Under these conditions, the predicted ACE-inhibitory activity of lizard fish hydrolysates was 84.45% and the predicted DH was 25.43%. To confirm the model’s validity, the experiment was performed at optimal conditions, in which the IP was 84% and the DH was 24%. These experimental values were in good agreement with the predicted value, confirming that these conditions were optimal for producing ACE-inhibitory peptides. 

### 2.3. Purification and Identification of ACE-Inhibitory Peptides

**Figure 2:**
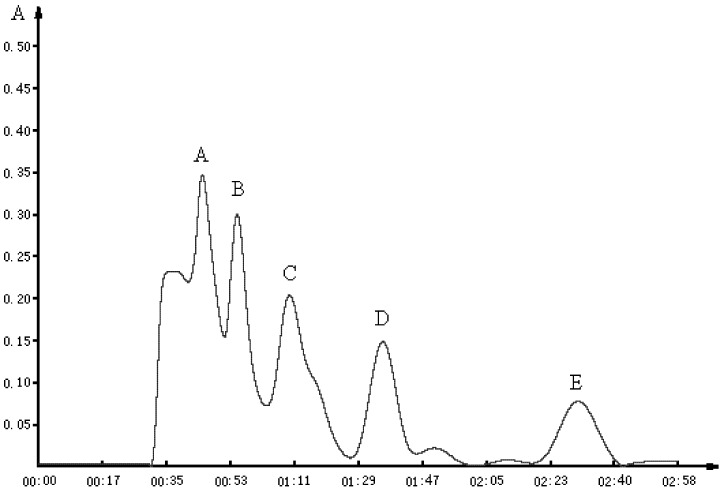
Chromatographic profile obtained by passing an aliquot of the fraction, smaller than 5000 Da, of lizard fish protein hydrolysate (LFPH-І) through a Sephadex G-15 column (1.6 cm × 45 cm). The column was eluted with water at a flow rate of 1 mL/min, and fraction C was found to possess the strongest activity.

**Figure 3:**
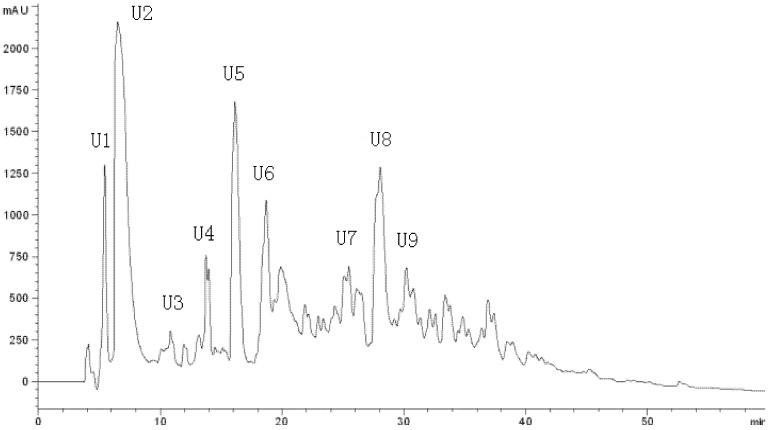
Chromatogram on a Hypersil ODS C18 column of the peptidic fraction from the active fraction C. The peak marked U7 was found to have the highest activity.

**Figure 4:**
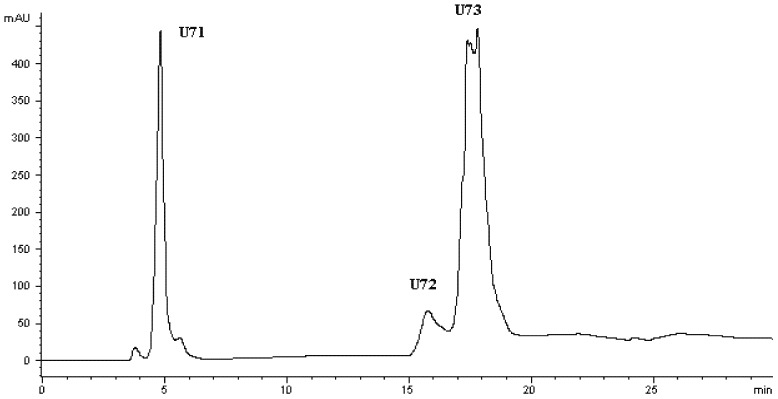
Chromatogram on a Hypersil ODS C18 column of the peptidic fraction from the active fraction U7. The peak marked U73 was found to have the highest activity.

**Figure 5:**
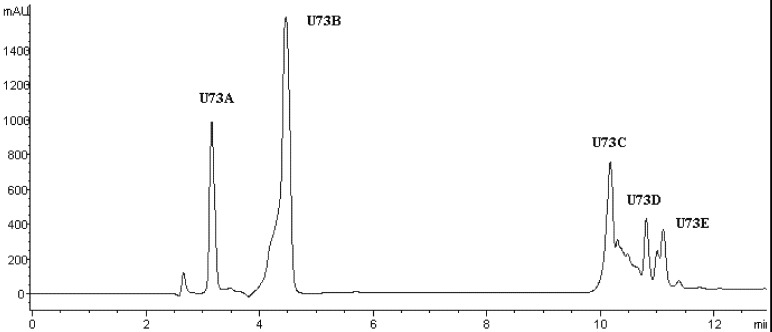
Chromatogram on a Zorbax SB C_18_ column of the peptidic fraction from the active fraction U73. The peak marked U73D was found to have the highest activity.

LFPH-І was fractionated by Sephadex G-15 chromatography into five portions: A, B, C, D, and E (Figure 2). Each fraction was measured for ACE-inhibitory activity, and fraction C was found to possess the strongest activity (Table 4). Active fraction C was purified by HPLC with the Hypersil ODS C_18_ columns (the first HPLC run). The fractions were pooled and lyophilized, and fraction U7 exhibited the strongest ACE-inhibitory activity (Table 4 and Figure 3). At the second HPLC step, the fraction U7 was further purified and divided into three portions (Figure 4), among which fraction U73 showed the strongest ACE-inhibitory activity (Table 4). To purify the strongest ACE-inhibitory peptide, fraction U73 was then applied to a Zorbax SB C_18_ column. As shown in Figure 5 and Table 4, the peak U73D showed the highest ACE-inhibitory activity and was applied to identify the amino acid sequence. The IC_50_ value of U73D was determined as 41 ± 1 µM.

**Table 4:** IP values of the fractions obtained from each separation step.

Fraction	IP (%)	Fraction	IP (%)	Fraction	IP (%)
A	83.18	U4	13.90	U73	78.92
B	86.95	U5	34.32	U73A	0
C	90.20	U6	68.72	U73B	0
D	77.46	U7	94.47	U73C	50.00
E	53.57	U8	49.05	U73D	84.05
U1	0	U9	75.72	U73E	64.95
U2	0	U71	0		
U3	11.83	U72	31.36		

### 2.4. Amino Acid Sequence Analysis

**Figure 6:**
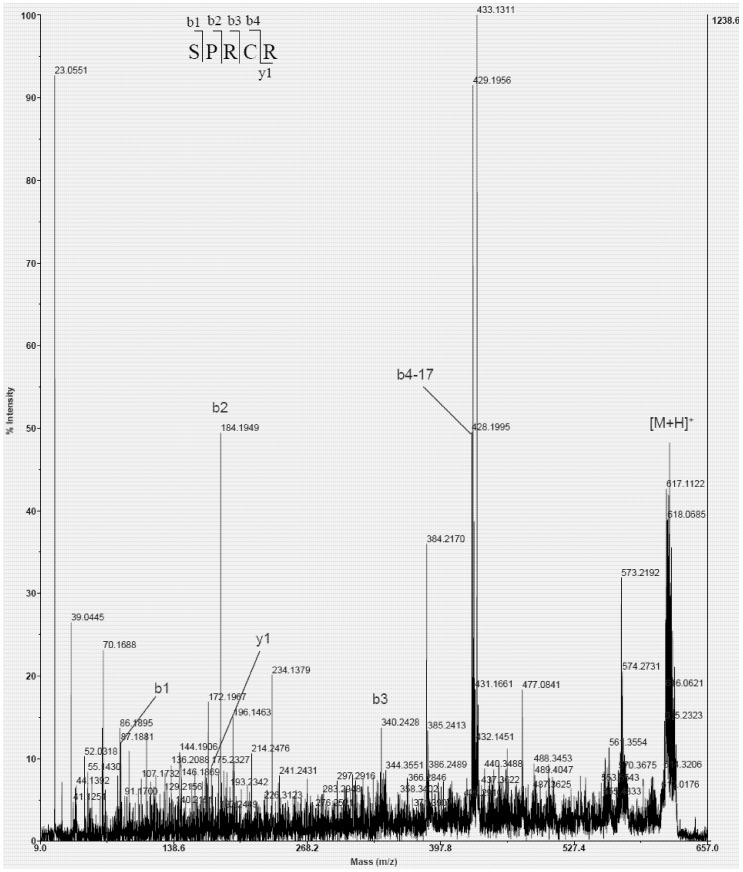
Peptide profile of peak U73D, *m/z* 618 performed by MALDI TOF/TOF mass spectrometry analysis.

The amino acid sequences of active fraction U73D were identified as Ser-Pro-Arg-Cys-Arg (SPRCR). As shown in Figure 6, the molecular mass (617 Da) corresponded with its sequence.

ACE-inhibitory peptides containing hydrophobic amino acids at each of the three *C*-terminal positions showed a strong ACE-inhibitory activity [22]. It was found that inhibitory peptides with arginine as the *C*-terminal residues have potent inhibitory activity and the positive charge of the side-chain group of arginine contribute to ACE inhibitory potency [23]. In the current study, the ACE-inhibitory peptide SPRCR, which is composed of five amino acid residues and possesses a hydrophobic residue, arginine as the *C*-terminal residue, exhibited strong ACE-inhibitory activity. Furthermore, this ACE-inhibitory peptide was synthesized to confirm the ACE inhibitory activity. The IC_50_ value of the synthesized peptide was 39 ± 1 µM, which corresponds to the IC_50_ value (41 ± 1 µM) of the natural peptide isolated by us. 

## 3. Experimental Section

### 3.1. Materials

The lizard fish was purchased from a local market in Nanning, China. Its muscle was rapidly separated. After being rinsed with deionized water, the removed muscle of the lizard fish was dried with hot air (90 °C) over 8 h, and then powdered. ACE (from rabbit lung; 2.0 units/mg of protein) and hippuryl-L-histidyl-L-leucine (HHL) were purchased from the Sigma Chemical Company (USA). Neutral protease was kindly provided by Nanning Pangbo Biological Engineering Co., Ltd. (China). By using casein as the substrate, the activity of neutral protease was measured by Measurement of Proteinase Activity (SB/T10317-1999, China) and found to have a value of 400,000 U/g. 

### 3.2. Enzymatic Hydrolysis

Under the conditions of E/S, pH, and temperature determined by the experimental design, lizard fish muscle protein was hydrolyzed with neutral protease. During the reaction of enzymatic hydrolysis, the pH was kept constant at the desired value by the addition of 0.1 M NaOH, and the volume of NaOH was recorded. After 2 h, the reaction was terminated by deactivating the enzyme at 95 °C in a water bath for 10 min. The pH was then adjusted to 7.0 by adding 0.1 M NaOH or 0.1 M HCl. The hydrolysate was centrifuged at 8000× *g* for 20 min (4 °C), and the supernates were lyophilized and used to measure ACE-inhibitory activity. 

### 3.3. Determination of the Degree of Hydrolysis

The degree of hydrolysis (DH) was estimated as the percentage of the peptide bonds cleaved during the enzymatic reaction (Equation 3) [24]:

DH% = B × N_b_ × (1/α)(1/M_p_) × (1/h_tot_) × 100 (3)

where B is the amount of NaOH consumed (mL); h_tot_ is the total number of peptide bonds in lizard fish muscle protein, assumed to be 7.836 eqv·g^−1^; N_b_ is the normality of NaOH, M_p_ is the mass of protein; and α is the average degree of dissociation of α-NH_2_ groups, calculated by the Equation 4:


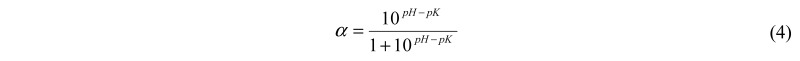


where p*K* is the average p*K* value of the α-amino groups liberated during hydrolysis. 

### 3.4. Measurement of ACE-Inhibitory Activity

The ACE-inhibitory activity of LFPH was determined by HPLC methods with some modification [25]. Briefly, for each assay, a sample solution (120 µL of 0.1 M sodium borate buffer containing 0.3 M NaCl at pH 8.3 or 120 µL of ACE inhibitor) with 30 µL of ACE solution (0.04 U/mL in 0.1 M sodium borate buffer containing 0.3 M NaCl at pH 8.3) was pre-incubated for 10 min at 37 °C. The mixture was incubated with 50 µL of substrate (5 mM HHL in 0.1 M sodium borate buffer containing 0.3 M NaCl at pH 8.3) for 60 min at the same temperature. The enzymatic reaction was terminated by the addition of 150 µL of 1 M HCl. The amount of hippuric acid released by the action of ACE was measured by HPLC on a Hypersil ODS C_18_ (4.0 mm × 250 mm, 5 μm, Agilent, Santa Clara, CA, USA) with 15% methanol containing 0.1% trifluoroacetic acid (TFA) at a flow rate of 1 mL/min. The absorbance was monitored at 228 nm. 

The inhibitory ratios were calculated by the following Equation 5:

IP (%) = [1 − (A_inhibitor_/A_control_)] × 100 (5)

where *IP* is the inhibitory ratio; A_inhibitor_ and A_control_ are the peak areas of the sample and the control (buffer added instead of test sample), respectively. IC_50_, the inhibitor concentration needed to inhibit 50% of enzyme activity, was determined by regression analysis of ACE inhibition (%) *versus* the log of the inhibitor concentration. 

### 3.5. Central Composite Rotatable Design (CCD) and Response-Surface Method

In the present study, the CCD of the three factors was used to optimize the enzymatic hydrolysis conditions of lizard fish muscle protein. Temperature (X_1_), E/S (X_2_), and pH (X_3_) were employed at five levels. The experimental designs are shown in Table 5. 

**Table 5:** Coded and decoded settings of the process parameters for lizard fish muscle protein hydrolysis, according to Central Composite Rotatable Design (CCD).

Factor	Level				
	−1.68 (−α)	−1	0	1	1.68 (+α)
X_1_: Temperature(°C)	40	44	50	56	60
X_2_: E/S(10000 U/g)	0.13	0.41	0.83	1.25	1.53
X_3_: pH	6.5	6.70	7.0	7.3	7.50


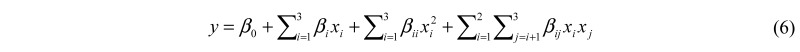


In the formula above (Equation 6), y (degree of hydrolysis or ACE-inhibitory activity in real value) is the response variable; x_i_ and x_j_ are independent variables; β_0_, β_i_, β_ii_, and β_ij_ are coefficients estimated by the model. The model evaluated the effect of each independent variable to the response. Statistical analysis was performed with Design Expert^®^ 7.0 (Stat-Ease Inc., Hennepin, MN, USA). A *P*-value of less than 0.05 was chosen for statistical significance.

### 3.6. Purification and Identification of ACE-Inhibitory Peptides

#### 3.6.1. Purification of ACE-Inhibitory Peptides

Lizard fish protein hydrolysate (LFPH) was ultrafiltrated with a 5000 MW cut-off filter (Labscale TFF System, Millipore Co., Billerica, MA, USA). The fraction LFPH-І, which was able to pass through the 5 kDa membrane, was lyophilized and used for further separation.

The fraction LFPH-І was fractionated using a Sephadex G-15 column (1.6 cm × 45 cm, Pharmacia Fine Chemicals, Uppsala, Sweden), which had been previously equilibrated with distilled water. The column was eluted with water at a flow rate of 1 mL/min and the elution was monitored at 280 nm. The fraction with the highest ACE-inhibitory activity was collected, lyophilized, and then purified by three-step HPLC. Elution peaks were monitored at 220 nm. Solvent A was 0.1% (v/v) TFA in water, and solvent B was 0.1% (v/v) TFA in acetonitrile. In the first HPLC step, separations were performed on a Hypersil ODS C_18_ column (4.0 mm × 250 mm, 5 μm, Agilent, Santa Clara, CA, USA) at a flow rate of 0.5 mL/min with a linear gradient of solvent B from 0% to 50% for 60 min. In the second HPLC step, the fractions showing the most efficient ACE-inhibitory activity were purified on a Hypersil ODS C_18_ (4.0 mm × 250 mm, 5 μm, Agilent, Santa Clara, CA, USA) with the gradient (5%–20% B, 8 min; 20%–30% B, 16 min) at a flow rate of 0.5 mL/min. The fraction with the highest ACE-inhibitory activity was further purified with Zorbax SB C_18_ (4.6 mm × 150 mm, 5 μm, Agilent, Santa Clara, CA, USA) and eluted with the gradient (5%–8% B, 5 min; 8%–27% B, 8 min; 27%–30% B, 12 min) at a flow rate of 0.5 mL/min. The fraction exhibiting the highest ACE-inhibitory activity was collected, lyophilized, and used to identify the amino acid sequence.

#### 3.6.2. Amino Acid Sequence Analysis

The amino acid sequence was identified using a 4800 Plus MALDI TOF/TOF™ Analyzer (Applied Biosystems, Beverly, MA, USA). It was performed at the School of Life Sciences and Technology, Guangxi University, Nanning, China. 

### 3.7. Synthesis of ACE-Inhibitory Peptide

The peptide was synthesized by GL Biochem Ltd, Shanghai, China.

## 4. Conclusion

A CCD was used to estimate the effects of temperature, E/S, and pH on response and factor interactions. The optimum operating conditions for enzymatic hydrolysis to achieve maximum ACE-inhibitory activity were a temperature of 48 °C, pH of 7.0, and E/S of 10,000 U/g. Furthermore, a novel ACE-inhibitory peptide was purified using ultrafiltration, gel filtration, and HPLC. The ACE-inhibitory peptide was identified and the peptide with sequence SPRCR was synthesized to confirm the ACE inhibitory activity. Therefore, it is to be expected that this peptide could be applied as a drug for preventing hypertension. However, further studies are being carried out to confirm its *in vivo* anti-hypertensive effects on animals. 
